# Diabetes Mellitus Type 2 and Coronary Artery Bypass Grafting: Elevated Risks of Mortality and Postoperative Complications

**DOI:** 10.1155/bmri/7509368

**Published:** 2026-07-10

**Authors:** Saad Al-Lahham, Areen Abu Hawash, Bayan Taha, Amal Zoghaier, Ali Kabha, Abeer Daraghmah, Maha Rabayaa, Mustafa Ghanim, Majdi Dweikat, Mohammad Abuawad, Malik Alqub

**Affiliations:** ^1^ Department of Biomedical Sciences, Faculty of Medicine and Allied Medical Sciences, An-Najah National University, Nablus, State of Palestine, najah.edu; ^2^ Department of Allied and Applied Medical Sciences, Faculty of Medicine and Allied Medical Sciences, An-Najah National University, Nablus, State of Palestine, najah.edu; ^3^ Department of Physiology, Faculty of Medicine, Bolu Abant İzzet Baysal University, Bolu, Türkiye

**Keywords:** CABG, glycemic control, mortality, postoperative complications, Type 2 diabetes mellitus

## Abstract

**Background:**

Type 2 diabetes mellitus (T2DM) has been associated with higher perioperative risks in coronary artery bypass grafting (CABG). This study examines preoperative characteristics, surgical factors, and postoperative outcomes of diabetic and nondiabetic patients undergoing CABG to identify differences in clinical profiles and complications.

**Methods:**

A retrospective analysis was conducted on 425 patients (214 nondiabetic, 211 diabetic) using SPSS 21. Continuous variables were compared using the Student *t*‐test, whereas categorical variables were compared using the chi‐squared test.

**Results:**

Diabetic patients had higher BMI (30.1 vs. 28.4 kg/m^2^, *p* < 0.001), worse glycemic control (HbA1c 7.9 vs. 5.6, *p* < 0.001), and higher rates of hypertension (84.4% vs. 62.6%, *p* < 0.001) and CHF (73% vs. 64%, *p* = 0.03). They also exhibited lower hemoglobin levels (12.8 vs. Thirteen.6 g/dL, *p* < 0.001), higher INR (1.0 vs. 0.9, *p* < 0.001), and higher rates of STEMI (13.3% vs. 5.1%, *p* = 0.003). Sex distribution also differed, with fewer males in the diabetic group (147 vs. 172, *p* = 0.007). Surgically, they required more urgent/emergent procedures (27% vs. 7.4%, *p* < 0.001) and longer bypass times (147.5 vs. 135.2 min, *p* = 0.024). Postoperatively, diabetics had higher mortality (5.2% vs. 0.5%, *p* = 0.003), stroke (13.3% vs. 2.8%, *p* < 0.001); cardiovascular events such as MI or stroke (9% vs. 0.5%, *p* < 0.001); infections (36.5% vs. 2.3%, *p* < 0.001); renal failure (20.4% vs. 2.8%, *p* < 0.001); gastrointestinal events (17.5% vs. 4.7%, *p* < 0.001); postoperative cardiac arrest (33.6% vs. 22%, *p* = 0.005); reoperation for bleeding (9.5% vs. 1.4%, *p* < 0.001); and longer hospital stays (9.2 vs. 6.3 days, *p* < 0.001). After adjustment for baseline demographic and clinical characteristics, diabetes mellitus was independently associated with significantly worse postoperative outcomes.

**Conclusion:**

Diabetic patients undergoing CABG had worse preoperative health, challenging surgical procedures, and much worse postoperative outcomes. These findings highlight the importance of targeted perioperative interventions for reducing hazards in this high‐risk population.

## 1. Introduction

Cardiovascular diseases (CVDs) remain the leading cause of death worldwide, with an estimated 17.9 million deaths per year (World Health Organization 2021) [1]. Coronary artery disease (CAD), a significant contributor to CVD‐related mortality, typically requires surgical intervention. Coronary artery bypass grafting (CABG) is among the most effective treatments for severe multivessel disease [2]. Diabetes mellitus (DM) is associated with a twofold to fourfold increased risk of cardiovascular complications postsurgery, despite advances in surgical procedures [3]. Diabetes affects an estimated 642 million people worldwide by 2040 [4]. Patients with DM undergoing CABG face an increased risk of mortality, stroke, and infections due to systemic metabolic dysfunction, microvascular damage, and impaired immune response [5]. Furthermore, diabetic patients frequently present with more complex CAD, requiring longer cardiopulmonary bypass (CPB) periods, all of which lead to delayed recovery and postoperative complications [6].

CABG remains an established treatment for patients with advanced multivessel CAD, and if performed at the appropriate time, it can play a significant role in lowering the death rate and problems associated with the condition. Despite the growing use of “off‐pump” CABG and interventional therapy, the CPB technique remains an important helping approach to CABG surgery [7].

Surgical stress in the use of CPB can cause various side effects, including systemic inflammatory response, nervous side effects, cardiac ischemia, renal failure, hemodynamic instability, and lung dysfunction. Several associated factors, including hypothermia, hemodilution, electrolyte imbalance, and pharmacological agents used during surgery with CPB, cause these complications [7]. As a result, individuals with T2DM are more likely to experience perioperative complications, including mortality. The 5‐year survival rate for diabetes patients after CABG was 85%, compared with 90% for nondiabetic patients. The impact of preoperative, intraoperative, and postoperative diabetes care, as well as perioperative hyperglycemia and hypoglycemia, on short‐term and long‐term operative outcomes remains a serious clinical concern with no universally acknowledged answer [8]. Although DM is additionally associated with other risk factors for CABG complications, such as obesity, hypertension, race, gender, and socioeconomic status, it may be a predictor of CABG concerns [9]. Even though T2DM is regarded as a key risk factor for the development of CVDs, the link between CVDs and T2DM is complex. Several studies found CVDs to be responsible for more than 80% of deaths in patients with T2DM. Unfortunately, the actual number of patients with T2DM is expected to double by the year 2030, indicating a spike in mortality during the following few years [10].

Given the rising prevalence of diabetes and its unfavorable impact on surgical outcomes, the goal of this study is to assess preoperative characteristics, intraoperative issues, and postoperative complications in diabetic versus nondiabetic patients undergoing CABG. By identifying crucial risk variables, we intend to create personalized perioperative therapies that will increase survival and recovery in this high‐risk population.

## 2. Methodology

### 2.1. Study Design and Setting

A retrospective cohort study design was used to investigate the impact of T2DM on complication rates of CABG and other associated risk factors in patients who underwent CPB. Preoperative, surgical, and postoperative variables were compared in two groups of patients who underwent CABG, with one group including patients with T2DM and the other group including nondiabetic patients. Participants′ data were obtained retrospectively from An‐Najah National University Hospital, which performs open‐heart surgeries. The hospitals were included in the study based on the availability of data in their recording systems.

### 2.2. Study Population

The study′s target population comprised individuals who had undergone CABG with the use of a CPB machine. The inclusion criteria involved adults aged more than 18 years or older with a history of CABG and complete medical records. Individuals with incomplete medical records are excluded from the study.

### 2.3. Sampling Procedure and Sample Size

The sample size was determined with a 5% margin of error and a 95% confidence interval using the Raosoft tool for calculating sample size (https://raosoftcalculator.net/). As a result, the study included a total of 425 participants who had undergone CABG, with 214 being nondiabetic and 211 being diabetic patients. The patients′ clinical records from the hospital′s computer systems were utilized to obtain clinical data.

### 2.4. Study Variables and Data Collection

Data is collected from electronic medical records, operative notes, and postoperative follow‐up records. A structured data collection tool was designed according to the literature to ensure consistency in information gathering. Trained personnel extracted relevant data, and a standard coding system was employed to maintain uniformity. The tool includes both quantitative and qualitative variables that capture a comprehensive dataset. This data encompasses demographics, comorbidities, surgical details, and postoperative outcomes.

The studied variables were categorized into three groups, including preoperative data, surgical‐related data, and postoperative data. The preoperative one included the patient′s demographic characteristics (i.e., age, gender, and BMI), some preoperative laboratory results (hemoglobin, HbA1c, INR, and creatinine), the left ventricular ejection fraction, smoking history, the presence of other chronic diseases, family history of CAD, previous medical interventions, and medication use. The surgical data included the surgical status, procedure‐related interventions, and duration. The postoperative data included the time to discharge from the hospital, postoperative laboratory findings, and the occurrence of postoperative complications.

### 2.5. Ethical Consideration

The study protocol, including the access and utilization of patient clinical data, followed the ethical guidelines outlined by the Declaration of Helsinki and was authorized by both the Institutional Review Board (IRB) (Ref. C.P.T. Jan. 2024/17) and local health authorities. The obtained information was exclusively employed for the study purposes; the confidentiality of the data was maintained and was not utilized beyond the scope of this study. All data were stored securely and anonymized during analysis.

### 2.6. Statistical Analysis

All statistical analyses were conducted using the Statistical Package for the Social Sciences Version 21 (SPSS 21) (IBM Corp., Armonk, New York, United States). The means and standard deviations (SD) were used to present continuous data, whereas frequencies and percentages were used for categorical variables. The study groups were compared using Student′s *t*‐test for the continuous variables and the chi‐square test for the categorical variables. The variables that were significant in the univariate analysis (*t*‐tests and chi‐square tests) were then introduced into the multivariate regression analysis (binary logistic regression was used for categorical variables and linear regression for continuous variables) to determine the effect of DM as an independent predictor for the dependent variables. In the multivariate model, the preoperative variables, including the demographic characteristics (age, gender, BMI, and smoking) and the other comorbidities and previous clinical interventions (family history of CAD, hypertension, dialysis, peripheral artery disease, CHF, prior CABG, and MI), were included. Variables were selected based on clinical relevance, and all were entered simultaneously using the enter method. Collinearity was assessed using tolerance and variance inflation factors, and there was no problematic collinearity. A *p* value of less than 0.05 was considered statistically significant.

## 3. Results

The preoperative characteristics of nondiabetic (*n* = 214) and diabetic (*n* = 211) patients revealed several significant differences. Diabetic patients had a higher BMI (30.1 vs. 28.4 kg/m^2^, *p* < 0.001), worse glycemic control (HbA1c 7.9 vs. 5.6, *p* < 0.001), and higher rates of hypertension (84.4% vs. 62.6%, *p* < 0.001) and CHF (73% vs. 64%, *p* = 0.03). They also exhibited lower hemoglobin levels (12.8 vs. Thirteen.6 g/dL, *p* < 0.001) and higher INR (1.0 vs. 0.9, *p* < 0.001). Notably, diabetics had lower rates of smoking (49.3% vs. 59.3%, *p* = 0.024) and prior CABG (4.3% vs. 9.8%, *p* = 0.02), but higher rates of STEMI (13.3% vs. 5.1%, *p* = 0.003). Sex distribution also differed, with fewer males in the diabetic group (147 vs. 172, *p* = 0.007). These findings highlight the distinct metabolic and cardiovascular profiles of diabetic patients presurgery (Table 1).

**Table 1:** Descriptive values (mean ± SD) or (%) of preoperative variables according to the study group.

Variables	Nondiabetic patients (*n* = 214)	Diabetic patients (*n* = 211)	*p*
Age (year) (mean ± SD)	63.7 ± 8.6	64.8 ± 8.4	0.186
Sex (m/f) (*n*)	172/42	147/64	**0.007**
BMI (kg/m^2^) (mean ± SD)	28.4 ± 4.0	30.1 ± 5.2	**< 0.001**
Left ventricular ejection fraction (mL) (mean ± SD)	50.4 ± 10.4	49.5 ± 9.9	0.372
HbA1c	5.6 ± 0.8	7.9 ± 1.5	**< 0.001**
INR	0.9 ± 0.3	1.0 ± 0.3	**< 0.001**
Hemoglobin	13.6 ± 2.1	12.8 ± 1.9	**< 0.001**
Creatinine	1.17 ± 0.6	1.24 ± 0.7	0.31
Smoker (%)	59.3	49.3	**0.024**
Family history of CAD (%)	31.8	31.8	0.54
Hypertension (%)	62.6	84.4	**< 0.001**
In dialysis (%)	14.5	10	0.1
Peripheral arterial disease (%)	10.3	14.7	0.109
CHF within 2 weeks (%)	64	73	**0.03**
Prior CABG (%)	9.8	4.3	**0.02**
Myocardial infarction (%)			
No	79	65.9	**0.003**
STEMI	5.1	13.3	
NSTEMI	15.9	20.8	
Previous cardiac intervention (%)	16.4	17.1	0.474
Medication use (%)	91.1	99.5	**< 0.001**

*Note:* The descriptive level of probability is determined by the Student′s *t*‐test for numerical variables and by the chi‐square test for categorical variables. Bolded *p*‐values indicate statistical significance (*p* < 0.05).

Abbreviations: CHF, congestive heart failure; NSTEMI, non‐ST elevation myocardial infarction; STEMI, ST elevation myocardial infarction.

Diabetic patients were less likely to undergo elective surgery (73% vs. 92.5%, *p* < 0.001) and more likely to require urgent (23.2% vs. 6.5%) or emergent (3.8% vs. 0.9%) procedures. Bypass time was longer in diabetics (147.5 vs. 135.2 min, *p* = 0.024), though aortic cross‐clamping time and LIMA use were comparable. The higher urgency and longer bypass times suggest greater surgical complexity in diabetic patients, potentially reflecting their poorer preoperative health status (Table 2).

**Table 2:** Descriptive values (mean ± SD) or (%) of surgical variables according to the study group.

Variables	Nondiabetic patients (*n* = 214)	Diabetic patients (*n* = 211)	*p*
Surgical status (%)			
Elective	92.5	73	**< 0.001**
Urgent	6.5	23.2	
Emergent	0.9	3.8	
LIMA use (%)	81.8	78.7	0.248
Assistive device (%)	15.9	10.4	0.064
Aortic cross‐clamping time (min) (mean ± SD)	93.2 ± 30.7	94.1 ± 29.1	0.757
Bypass time (min) (mean ± SD)	135.2 ± 46.3	147.5 ± 64.3	**0.024**

*Note:* The descriptive level of probability is determined by the Student′s *t*‐test for numerical variables and by the chi‐square test for categorical variables. Bolded *p*‐values indicate statistical significance (*p* < 0.05).

Abbreviation: LIMA, left internal mammary artery.

Diabetic patients had a significantly longer time to discharge (9.2 vs. 6.3 days, *p* < 0.001), reflecting greater postoperative complications. Hemoglobin levels were higher in diabetics postsurgery (10.3 vs. 9.5 g/dL, *p* < 0.001), possibly due to differences in fluid management or transfusion practices. INR levels were comparable (1.32 vs. 1.29, *p* = 0.53), but creatinine levels were elevated in diabetics (1.4 vs. 1.1 mg/dL, *p* < 0.001), indicating poorer renal function. Mortality was drastically higher in diabetics (5.2% vs. 0.5%, *p* = 0.003). Permanent stroke (13.3% vs. 2.8%, *p* < 0.001) and cardiovascular events such as MI or stroke (9% vs. 0.5%, *p* < 0.001) were also more frequent, highlighting neurological vulnerabilities. Infective complications (36.5% vs. 2.3%, *p* < 0.001) and pneumonia (24.2% vs. 1.4%, *p* < 0.001) were strikingly more common in diabetics, underscoring their impaired immune response. Renal failure (20.4% vs. 2.8%, *p* < 0.001) and gastrointestinal events (17.5% vs. 4.7%, *p* < 0.001) further demonstrated multisystem dysfunction. Reoperation for bleeding occurred more often in diabetics (9.5% vs. 1.4%, *p* < 0.001), suggesting impaired hemostasis or surgical challenges. Postoperative cardiac arrest (33.6% vs. 22%, *p* = 0.005) was also elevated, though atrial fibrillation rates were similar (32.2% vs. 29%, *p* = 0.267). Multisystem failure showed a nonsignificant trend toward higher incidence in diabetics (2.8% vs. 0.5%, *p* = 0.059) (Table 3).

**Table 3:** Descriptive values (mean ± SD) or (%) of postoperative variables according to the study group.

Variables	Nondiabetic patients (*n* = 214)	Diabetic patients (*n* = 211)	*p*
Time to discharge (day) (mean ± SD)	6.3 ± 1.9	9.2 ± 6.0	**< 0.001**
Hemoglobin (g/dL) (mean ± SD)	9.5 ± 2.1	10.3 ± 2.3	**< 0.001**
INR (mean ± SD)	1.29 ± 0.4	1.32 ± 0.5	0.53
Creatinine (mg/dL) (mean ± SD)	1.1 ± 0.7	1.4 ± 1.1	**< 0.001**
Mortality (%)	0.5	5.2	**0.003**
Any permanent stroke (%)	2.8	13.3	**< 0.001**
Infective complications (%)	2.3	36.5	**< 0.001**
Pneumonia (%)	1.4	24.2	**< 0.001**
Renal failure (%)	2.8	20.4	**< 0.001**
Gastrointestinal events (%)	4.7	17.5	**< 0.001**
Reoperation for bleeding (%)	1.4	9.5	**< 0.001**
Postoperative cardiac arrest (%)	22	33.6	**0.005**
Cardiovascular accident (%)	0.5	9	**< 0.001**
Multisystem failure (%)	0.5	2.8	0.059
Postoperative atrial fibrillation (%)	29	32.2	0.267

*Note:* The descriptive level of probability is determined by the Student′s *t*‐test for numerical variables and by the chi‐square test for categorical variables. Bolded *p*‐values indicate statistical significance (*p* < 0.05).

### 3.1. Multivariate Regression Analysis of the Independent Predictive Role of DM in Postoperative Variables

After adjustment for baseline demographic and clinical characteristics, DM was independently associated with significantly worse postoperative outcomes. Diabetes was associated with prolonged time to discharge (*B* = 2.6, *p* < 0.001; 95% CI: 1.68–3.53). Postoperative creatinine levels were significantly higher (*B* = 0.12, *p* = 0.017; 95% CI: 0.02–0.21). In addition, diabetic patients had significantly longer CPB times (*B* = 15.29 min, *p* = 0.009; 95% CI: 3.9–26.68).

Concerning clinical outcomes, diabetes was significantly associated with increased mortality (OR = 16.41, 95% CI: 1.92–139.9; *p* = 0.011). Diabetic patients also had higher odds of major postoperative complications, including: any permanent stroke (OR = 4.44, 95% CI: 1.68–11.7; *p* = 0.003), infection‐related complications (OR = 19.27, 95% CI: 7.38–50.34; *p* < 0.001), pneumonia (OR = 24.77, 95% CI: 7.31–83.99; *p* < 0.001), renal failure (OR = 20.23, 95% CI: 6.25–65.51; *p* < 0.001), gastrointestinal events (OR = 5.51, 95% CI: 2.45–12.42; *p* < 0.001), reoperation for bleeding (OR = 8.4, 95% CI: 2.28–31.03; *p* = 0.001), postoperative cardiac arrest (OR = 1.74, 95% CI: 1.08–2.79; *p* = 0.023), cardiovascular accident (OR = 16.45, 95% CI: 2.08–130.09; *p* = 0.008). Overall, diabetes remained an independent predictor of increased perioperative morbidity and mortality after controlling for potential confounders (Table 4).

**Table 4:** Multivariate logistic regression analysis using diabetes mellitus as an independent predictor for the postoperative effects.

Dependent variable	*B*	*p*	Exp(*B*)	95% C.I.	
				Lower	Upper
Time to discharge (days)	2.6	< 0.001	0.28	1.68	3.53
Hemoglobin postoperation (g/dL)	−0.37	0.074	−0.088	−0.77	0.04
Creatinine postoperation (mg/dL)	0.12	0.017	0.09	0.02	0.21
Bypass time (min)	15.29	0.009	0.14	3.9	26.68
Mortality	2.8	0.011	16.41	1.92	139.9
Any permanent stroke	1.49	0.003	4.44	1.68	11.7
Infective complications	2.96	< 0.001	19.27	7.38	50.34
Pneumonia	3.21	< 0.001	24.77	7.31	83.99
Renal failure	3.01	< 0.001	20.23	6.25	65.51
Gastrointestinal event	1.71	< 0.001	5.51	2.45	12.42
Reoperation for bleeding	2.13	0.001	8.4	2.28	31.03
Postoperative cardiac arrest	0.55	0.023	1.74	1.08	2.79
Cardiovascular accident	2.8	0.008	16.45	2.08	130.09

## 4. Discussion

The current study was conducted on a representative sample of patients who received CABG, which is now considered the optimal revascularization procedure for diabetic patients [11, 12]. Diabetic and nondiabetic patients were almost equally represented in this study. This balance establishes a beneficial platform for comparing outcomes between the two groups. The gender pattern, with a higher proportion of males compared with females, is consistent with prior research that found a higher frequency of cardiovascular illnesses and related procedures in males compared with females [13–15]. This gender disparity is often attributed to the protective effects of estrogen in premenopausal women and the higher prevalence of risk factors such as smoking and hypertension in men [16, 17]. Diabetic patients were slightly older and had a higher BMI, which is a known risk factor for complications during and after surgery [18–21]. The higher hemoglobin A1C levels in diabetic patients indicate poorer glycemic control, which is associated with increased surgical risks [22–26]. Notably, diabetic patients had higher rates of hypertension and congestive heart failure, which are known comorbidities of diabetes and contribute to worse surgical outcomes [27, 28].

Diabetic patients underwent fewer elective surgeries and more urgent and emergent procedures, suggesting that their disease state might necessitate more immediate surgical intervention. A longer bypass time is associated with a higher risk of complications such as myocardial injury [29], stroke [30], and renal dysfunction [31–33]. Studies have demonstrated that extended bypass times are linked to higher mortality and morbidity rates post‐CABG [34, 35]. These findings are critical as they highlight the need for tailored surgical planning and intervention strategies for diabetic patients. The current study found that mortality was as high as 11.8% in diabetic patients [36]. Diabetic patients exhibited a higher BMI and worse glycemic control than nondiabetic patients, consistent with known metabolic derangements in diabetes [3]. The higher prevalence of hypertension and congestive heart failure in diabetics aligns with studies linking diabetes to accelerated CVD [10]. Notably, lower hemoglobin levels may reflect diabetic nephropathy or chronic inflammation [5].

Our adjusted analysis revealed that diabetes patients had much lower recovery characteristics than nondiabetics. Diabetes was particularly associated with prolonged hospital stays. Diabetics experienced higher CPB times and delayed discharge (time‐to‐discharge increased by approximately 2.6 days). This is consistent with recent data that surgical patients with diabetes frequently have long hospitalizations and ICU stays due to their comorbidities [37]. Postoperative hyperglycemia is associated with worse clinical outcomes, including a higher rate of acute kidney injury, delirium, and pulmonary infection, greater duration of mechanical ventilation, length of ICU stay, length of hospitalization, and higher rate of automatic discharge or death [38]. Studies on intraoperative and postoperative medical intervention to regulate glycemia in cardiac surgery patients demonstrate improved short‐term medical outcomes, including decreased mortality and improved glycemic control, reduction in infection rates, and shorter length of stay [39]. In other studies, postoperative outcomes were significantly worse in diabetic patients with prolonged hospitalization in these patients [40, 41].

Diabetes strongly predicts organ‐system complications. We found that diabetics had much greater rates of renal failure and infection. Diabetic patients exhibited greater serum creatinine levels and a significantly higher risk of acute renal failure following surgery. This is consistent with the well‐known fragility of diabetic kidneys. In a meta‐analysis of surgical cohorts, five studies found renal failure. The pooled OR of renal insufficiency in patients with DM versus those without DM was 1.987 (1.311, 3.013), indicating that DM was a risk factor for postoperative renal insufficiency, implying that even mild diabetic nephropathy can lead to significant acute kidney injury under surgical stress [37]. In another study, Wang et al. found that independent of baseline renal function or cardiac function, DM was associated with an increased risk of acute kidney injury after CABG, especially in patients with insulin treatment, who also had a higher severity of acute kidney injury [42].

Patients with diabetes were also more likely to suffer from infectious diseases. Diabetics had a much increased risk of postoperative infection and pneumonia. This is consistent with published reports; diabetic surgery patients are known to have a much higher infection risk. For instance, diabetics experience nearly double the incidence of postoperative pneumonia across various procedures [43]. In part, the increase is due to immune dysfunction and microvascular damage in diabetes. Chronic hyperglycemia can compromise neutrophil function and wound healing, increasing the risk of pneumonia and surgical site infections. The main reasons for the predisposition to the cited infections are due to the strong association between DM and angiopathy, neuropathy, and hyperglycemia [44]. Diabetic patients present an increased risk for postoperative infections due to impaired host defense mechanisms, such as impaired wound healing and granulocyte function, decreased cellular immunity, impaired complement function, and reduced immune response, which may be influenced by glycemic control [45, 46].

According to the stated meta‐analysis, diabetes increases the risk of postoperative infection. Similarly, wound healing problems and hematomas were more common in individuals with diabetes in those investigations. Our series′ high infection rates (particularly pneumonia and systemic infections) are completely consistent with the literature. Overall, the combination of inadequate immune response and vascular dysfunction in diabetes explains the spike in infection‐related and inflammatory consequences [37, 43].

Diabetic patients additionally scored poorly on cardiovascular and neurologic outcomes. We found a greater risk of postoperative stroke and other cardiovascular events. Previous studies indicated that individuals with diabetes undergoing surgical and percutaneous cardiac operations, compared with people without diabetes, are at higher risk of mortality, reinterventions, and sequelae like deep sternal wound infections, stroke, and kidney failure [47–51]. This is scientifically possible because diabetes promotes atherosclerosis and affects endothelial function, making patients more likely to experience thrombotic or embolic events after surgery. Consistent with these pathways, our diabetic sample experienced considerably more strokes and cardiac arrests than controls.

Most dramatically, diabetes has been associated with a significantly higher risk of death. Our results indicated a positive association between having diabetes and mortality, suggesting that diabetes has a significant mortality disadvantage following surgery. However, the low number of mortality cases in the studied sample might limit our interpretation of the size effect, and further studies should be conducted on a larger sample size to determine the size effect of diabetes on mortality. Likewise, the heart surgery literature consistently reveals that diabetics have higher short‐ and long‐term mortality than nondiabetics. In other words, even after adjusting for age, comorbidities, and procedural variables, diabetes status predicts poor survival. Diabetics′ higher death rate is most likely due to their increased load of end‐organ damage (heart disease, kidney disease, etc.) and poorer tolerance to surgical stress [52]. Recent studies by Szabo et al. [53] and Kubal et al. [54] concur with our findings, reporting an intrahospital mortality of 2.6% and 2.4% for their DM patient populations, respectively. Bundhun et al. reported consistent results with meta‐analyses reporting a 2–3‐fold increase in mortality post‐CABG [55]. In our analysis, patients with DM presented more comorbidities, such as more severe atherosclerotic disease and more complications. Therefore, they had a more urgent indication for surgery and a significantly higher usage of extracorporeal circulation, which can contribute to a greater chance of complications after surgery.

## 5. Conclusion

This study demonstrates the considerable impact of DM on perioperative risks and postoperative outcomes in patients having CABG. Diabetic patients had worse preoperative health profiles, including a higher BMI, poorer glycemic control, and a higher incidence of hypertension and congestive heart failure. These characteristics contributed to more difficult surgical treatments, as shown by longer CPB periods and a higher prevalence of urgent/emergent surgeries. Diabetic individuals had significantly worse postoperative outcomes, including an increased risk of stroke, infections, renal failure, and longer hospital admissions. These findings highlight diabetes patients′ increased vulnerability to multisystem problems after CABG, which are most likely caused by metabolic inefficiency, microvascular damage, and compromised immunological responses. The study stresses the crucial necessity for specialized perioperative treatments for diabetes patients, such as strict glucose control, optimal surgical planning, and diligent postoperative monitoring, to reduce hazards. Future study should look into tailored interventions to enhance outcomes in this high‐risk population, especially in resource‐limited settings.

### 5.1. Limitations

When assessing the results, it is important to point out several study limitations. First, the retrospective methodology reduces the ability to account for all confounding factors, making causal connections between diabetes status and postoperative outcomes unlikely. Standardized and documented perioperative variables, including fluid management and transfusion decision‐making, were lacking. Transfusion and intravenous fluid delivery may have affected postoperative hematologic parameters, including hemoglobin levels. Thus, higher postoperative hemoglobin levels in diabetic patients may indicate treatment variability rather than diabetes. Second, diabetes and nondiabetics had statistically significant but modest differences in preoperative INR values. Both values were within the normal reference range; thus, the divergence is unlikely to be clinically significant. This finding should be interpreted cautiously since the statistical significance may be due to the sample size rather than a clinically relevant coagulation status change. Moreover, as with many retrospective clinical investigations, unmeasured perioperative factors and institutional practice variations may have affected the results. Finally, the interpretation of the very large adjusted odds ratios for outcomes such as mortality, infection, pneumonia, and renal failure should remain cautious given the wide confidence intervals. Future prospective studies using standardized perioperative treatment regimens are needed to better understand diabetes and postoperative hematologic and clinical outcomes.

## Author Contributions

Saad Al‐Lahham and Malik Alqub conceptualized and supervised the study. Areen Abu Hawash, Bayan Taha, Amal Zoghaier, Ali Kabha, and Abeer Daraghmah participated in data collection and data curation. Mustafa Ghanim contributed to methodology development and data interpretation. Maha Rabayaa, Majdi Dweikat, and Mohammad Abuawad performed the statistical analysis and assisted in manuscript preparation. Saad Al‐Lahham, Mohammad Abuawad, and Malik Alqub drafted the manuscript.

## Funding

No funding was received for this manuscript.

## Disclosure

All authors critically revised the manuscript, approved the final version, and agreed to be accountable for all aspects of the work.

## Consent

The authors give the Publisher the authors′ permission to publish the work.

## Conflicts of Interest

The authors declare no conflicts of interest.

## Data Availability

Data are available on request due to privacy/ethical restrictions.
